# Measurement of FGFR3 signaling at the cell membrane *via* total internal reflection fluorescence microscopy to compare the activation of *FGFR3* mutants

**DOI:** 10.1016/j.jbc.2022.102832

**Published:** 2022-12-27

**Authors:** Ingrid Hartl, Veronika Brumovska, Yasmin Striedner, Atena Yasari, Gerhard J. Schütz, Eva Sevcsik, Irene Tiemann-Boege

**Affiliations:** 1 Institute of Biophysics, Johannes Kepler University, Linz, Austria; 2 Insitute of Applied Physics, TU Wien, Vienna, Austria

**Keywords:** receptor tyrosine kinase, fibroblast growth factor, signaling, total internal reflection fluorescence microscopy, GRB2, FGFR3, ACH, achondroplasia, EC, extracellular, FGFR, fibroblast growth factor receptor, FRS2α, FGFR substrate 2α, GRB2, growth factor receptor–bound 2, HCH, hypochondroplasia, IC, intracellular, mGFP, monomeric GFP, mRFP, monomeric red fluorescent protein, RTK, receptor tyrosine kinase, TD1, thanatophoric dysplasia I, TDII, thanatophoric dysplasia II, TIRF, total internal reflection fluorescence, TM, transmembrane, SP, signal peptide

## Abstract

Fibroblast growth factor receptors (FGFRs) initiate signal transduction *via* the RAS/mitogen-activated protein kinase pathway by their tyrosine kinase activation known to determine cell growth, tissue differentiation, and apoptosis. Recently, many missense mutations have been reported for *FGFR3*, but we only know the functional effect for a handful of them. Some mutations result in aberrant FGFR3 signaling and are associated with various genetic disorders and oncogenic conditions. Here, we employed micropatterned surfaces to specifically enrich fluorophore-tagged FGFR3 (monomeric GFP [mGFP]-FGFR3) in certain areas of the plasma membrane of living cells. We quantified receptor activation *via* total internal reflection fluorescence microscopy of FGFR3 signaling at the cell membrane that captured the recruitment of the downstream signal transducer growth factor receptor–bound 2 (GRB2) tagged with mScarlet (GRB2-mScarlet) to FGFR3 micropatterns. With this system, we tested the activation of FGFR3 upon ligand addition (fgf1 and fgf2) for WT and four FGFR3 mutants associated with congenital disorders (G380R, Y373C, K650Q, and K650E). Our data showed that ligand addition increased GRB2 recruitment to WT FGFR3, with fgf1 having a stronger effect than fgf2. For all mutants, we found an increased basal receptor activity, and only for two of the four mutants (G380R and K650Q), activity was further increased upon ligand addition. Compared with previous reports, two mutant receptors (K650Q and K650E) had either an unexpectedly high or low activation state, respectively. This can be attributed to the different methodology, since micropatterning specifically captures signaling events at the plasma membrane. Collectively, our results provide further insight into the functional effects of mutations to FGFR3.

As a family of cell surface receptors, receptor tyrosine kinases (RTKs) initiate intracellular (IC) signaling cascades that activate or inhibit different transcription factors linked to numerous cellular processes, such as cell growth, development, and apoptosis (1). Although different RTKs activate the same IC signaling pathways, the cellular response can be diverse. Fibroblast growth factor receptors (FGFRs) are a subclass of RTKs and comprise four members (2) that are evolutionary highly conserved across multicellular species (1, 3). FGFRs are activated by binding of their ligand fgf, which typically involves receptor dimerization (4), although formation of larger clusters has been observed for FGFR1 in a ligand-specific manner, suggesting that the FGFR oligomerization state mediates cellular responses to different ligands (5). Activation of the receptor leads to multiple transautophosphorylation events of several tyrosine residues in the tyrosine kinase domain (residues Y577, Y579, Y647, Y648, and Y760 in FGFR3) (4). The phosphorylated tyrosines are docking sites for IC downstream signaling proteins containing Src homology-2 and phosphotyrosine-binding domains. Specifically, activated FGFRs phosphorylate and activate the membrane-anchored adaptor protein constitutively associated with the receptor, FGFR substrate 2α (FRS2α), which then binds the growth factor receptor–bound 2 (GRB2) and Src homology-2–containing protein tyrosine phosphatase 2. GRB2 then recruits Son of Sevenless and GRB2-associated-binding protein 1.

In addition, FGFRs are able to interact with multiple ligands of the fgf family inducing different levels of activation, which adds yet another layer of complexity to FGFR signaling (6, 7). In particular, the splice form FGFR3c used in this study is mainly activated by fgf1, fgf2, fgf8, and fgf9 (8, 9). Of these ligands, fgf1 and fgf2 were reported to show differences in inducing kinase phosphorylation with fgf2 having a stronger activating effect when added at saturating conditions (10). The resulting outcome of the receptor's signaling cascade is not only determined by the interplay of different components of the signaling pathways but also by the number of phosphorylated docking sites in the tyrosine kinase domain, time of activation, and time of inactivation, etc. (1). Once FGFR activation has taken place, the signaling cascade needs to be silenced. One common mechanism for signal downregulation is endocytosis that removes the receptor from the plasma membrane. Internalized receptors can be recycled back to the cell surface, rendering them unavailable only for a limited time, or, alternatively, they are permanently eliminated by lysosomal degradation after internalization (11). Receptor inactivation is initiated by E3 ubiquitin–protein ligase CBL that forms a complex with phosphorylated FRS2α and GRB2 leading to the ubiquitination of FGFRs and FRS2, which is the signal for receptor internalization (4, 11).

Furthermore, single-point mutations that cause missense amino acid substitutions have been reported to increase the receptor’s activity also in the absence of ligand and have been defined as gain-of function mutations with profound phenotypic effects causing multiple genetic disorders even in the heterozygous state (reviewed in Refs. (12, 13, 14, 15)). Several missense variants of *FGFR3* have been documented in genetic studies of patients with phenotypic disorders, such as achondroplasia (ACH), thanatophoric dysplasia I (TDI) and II (TDII), Muenke syndrome, hypochondroplasia (HCH), among others reported in the Human Gene Mutation database and also in tumors listed in the Catalogue Of Somatic Mutations In Cancer (16). Furthermore, *FGFR3* has been categorized as a cancer driver gene involved in cell survival with a 99% oncogene score (17, 18, 19). Over 7% of the sequenced tumors were positive for *FGFR3* mutants, some of them affecting the same codon resulting in different amino acid substitutions (cancer.sanger.ac.uk, (16)), further highlighting the proliferative properties of *FGFR3* mutations. Given that FGFR3 is mainly expressed in the cartilage, brain, lung, and the spinal cord (20, 21), it appears to be most important in the regulation of cartilage growth, being a physiologic negative regulator of chondrocyte proliferation, thus restricting skeletal growth (22, 23). In addition, signaling is tightly regulated by interactions between epithelial and mesenchymal cells during organogenesis as well as the initiation and proximal–distal growth of the limb bud (23). Thus, *FGFR3* mutations are mainly associated with chondrodysplasia syndromes (4, 22, 24).

In this work, we focused on four different substitutions associated with chondrodysplasia syndromes of different severity: K650Q (c.1948A>C, HCH) (25, 26); G380R (c.1138G>A, ACH) (27, 28, 29); Y373C (c.1118A>G, TDI) (30); and K650E (c.1948A>G, TDII) (31). It has been reported that K650E causes a higher basal level of tyrosine phosphorylation than the G380R substitution, providing a possible biochemical explanation why the phenotype of TDII is more severe than that of ACH (25, 32, 33).

In spite of the strong phenotypic consequences of *FGFR3* mutations, not much is known how substitutions affect the receptor’s signaling. Only a few mutations have been validated with experimental data in terms of the effect of the mutation. These include functional studies that experimentally examined the effect of the mutant protein on receptor activation on its signaling (10, 25, 34, 35). The potential deleteriousness (both gain- or loss-of-function modifications) of some mutations is also predicted by *in silico* analysis such as combined annotation–dependent depletion or SIFT scores or by merging information from multiple component methods with some experimental data (36, 37). However, information on the effect of mutations on the protein's function is still scarce.

For this reason, the purpose of this work was to implement an assay to determine the activity of FGFR3 in living cells that can be further used in the analysis of mutants. Currently, the main method to quantify the activation of FGFRs is Western blotting, which determines the phosphorylation state of tyrosines in the adaptor protein docking sites and compares the signal of the phosphorylated protein to the signal intensity of the pan-protein (25, 33, 38, 39) or the *in vitro* kinase assay, which uses immunoprecipitated cell lysates that are incubated with ^32^P-ATP (25). These methods are labor intensive and have other potential pitfalls like poor antibody specificity and electrophoretic resolution, as reviewed in Ref. (40). Moreover, they measure the protein in whole-cell lysates.

In view of the limitations presented by methodologies relying on whole-cell lysates, we here chose to employ a protein micropatterning approach (41, 42, 43) to determine the kinase activity of FGFR3 directly in living cells. In this assay, cells are grown on microstructured surfaces featuring regular arrays of antibodies against the protein of interest (“bait”) with the recruitment of a fluorescently labeled interaction partner (“prey”) being monitored *via* fluorescence microscopy. The interaction strength between the two proteins can then be quantified *via* the signal contrast between the prey signal intensity within and outside the bait regions (Fig. 1*A*) (42, 43). In the present study, FGFR3 served as prey, and the colocalization of the downstream adaptor protein GRB2 was taken as a measure for the kinase activity of FGFR3 upon stimulation. This experimental design allows to specifically detect only signaling events of the mature protein at the plasma membrane and has been used before to study RTK signaling hubs of the epidermal growth factor receptor (41) and kinase recruitment to the T-cell receptor signaling complex (44).

**Figure 1:**
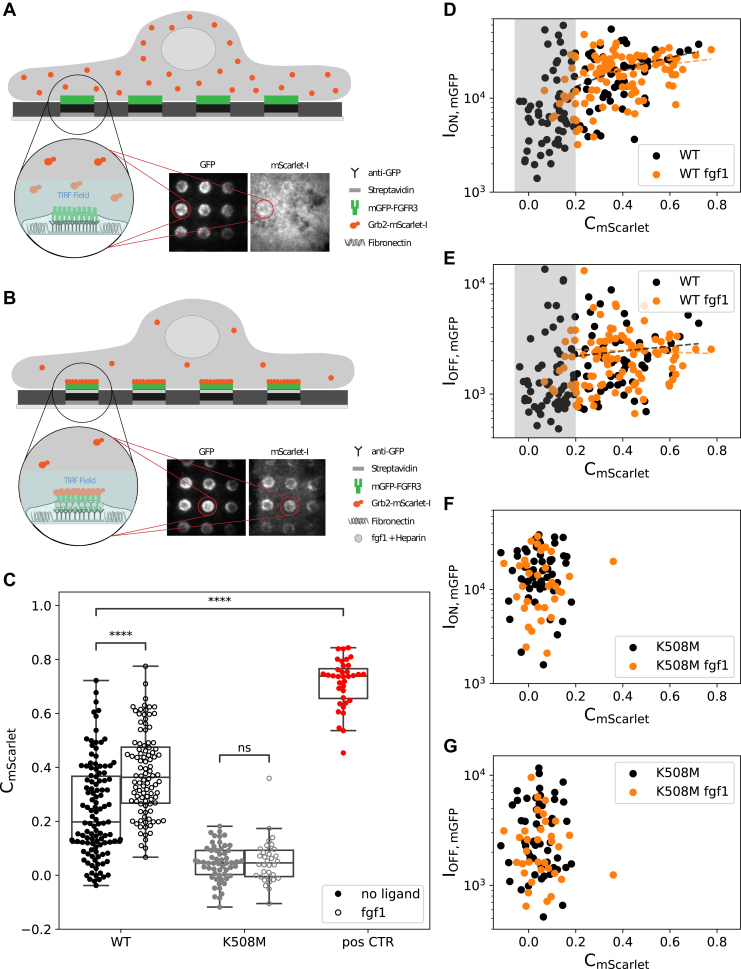
**Experimental design and proof of principle.***A* and *B,* antibody patterns are used to enrich and immobilize mGFP-FGFR3 at specific sites (“ON” regions) in the plasma membrane of HeLa cells, leaving other regions depleted of mGFP-FGFR3 (“OFF”). Colocalization of the adaptor protein GRB2-mScarlet to mGFP-FGFR3 patterns reports on the activation state of FGFR3, with no or little copatterning observable in the nonactivated state (*A*) and a high degree of copatterning for the activated receptor after addition of the ligand fgf1 (*B*). TIRF illumination is used to specifically detect membrane-proximal protein. *C,* the fluorescence contrast of GRB2-mScarlet (C_mScarlet_) relates the fluorescence intensity within ON (I_ON,mGFP_) and OFF (I_OFF,mGFP_) areas of FGFR3-enriched regions and serves to quantify the extent of colocalization. Each dot represents one cell. C_mScarlet_ data for the WT receptor, a kinase-dead mutant (K508M) and an mGFP-FGFR3-mScarlet fusion protein as positive control is shown (*p* value annotation legend: ∗0.01 ≤ *p* ≤ 0.05; ∗∗0.001 ≤ *p* ≤ 0.01; ∗∗∗0.0001 ≤ *p* ≤ 0.001; and ∗∗∗∗*p* ≤ 0.0001). *D* and *E,* correlation between the receptor’s intensity in ON (*D*) and OFF (*E*) regions and the GRB2-mScarlet contrast for the WT receptor. Data in the absence (*black*) and presence (*orange*) of fgf1 are shown. The *gray box* indicates the cell population with C_mScarlet_ <0.2, which likely represents nonactivated cells. *F* and *G,* correlation between GRB2-mScarlet contrast and mGFP-FGFR3 intensity in ON (*F*) and OFF (*G*) regions for K508M. All correlation coefficients can be found in Table S1. FGFR3, fibroblast growth factor receptor 3; GRB2, growth factor receptor–bound 2; mGFP, monomeric GFP; TIRF, total internal reflection fluorescence.

Using this micropatterning approach, we quantified the activation of WT FGFR3 as well as four different pathogenic mutants associated with congenital disorders. Specifically, we focused on two mutations in the transmembrane (TM) domain and flanking regions (G380R and Y373C) reported to increase receptor dimerization (45) and sustained extracellular signal–regulated kinase activation (35) and two mutations in the kinase domain activation loop (K650Q and K650E).

## Results

### Characterization of the experimental system

In order to measure FGFR3 activation, HeLa cells expressing monomeric GFP (mGFP)-FGFR3 and GRB2-mScarlet were seeded onto microstructured surfaces featuring regular patterns of a monoclonal antibody against mGFP (42, 46). The mGFP-FGFR3 at the plasma membrane was enriched and immobilized according to these micropatterns, with the fluorescence intensity reporting on the extent of mGFP-FGFR3 enrichment (I_ON,mGFP_), leaving other regions depleted of mGFP-FGFR3 (yielding I_OFF,mGFP_). The corecruitment of GRB2-mScarlet to mGFP-FGFR3 patterns was imaged using total internal reflection fluorescence (TIRF) microscopy (Fig. 1, *A* and *B*) with the fluorescence contrast, $C=\frac{I_{ON}-I_{OFF}}{I_{ON}-I_{bg}}$ serving as a measure for the activation.

First, we determined the level of GRB2-mScarlet recruitment to the mGFP-FGFR3 WT construct in the nonliganded state. We observed a pronounced pattern formation in the mGFP channel, reported here as the mean fluorescence contrast for mGFP (mean C_mGFP_ = 0.85 ± 0.01, Table 1). While ∼50% of the cells showed a rather uniform distribution of GRB2-mScarlet, the other half exhibited a low to moderate fluorescent contrast for GRB2-mScarlet, yielding a mean C_mScarlet_ = 0.24 ± 0.02 when considering the total number of cells (Fig. 1*C* and Table 1). However, when activating the cells expressing the WT receptor with 50 ng/ml of ligand fgf1 (4, 8) and 1 μg/ml of the cofactor heparin (47), we observed a significant increase in GRB2-mScarlet contrast (mean C_mScarlet_ = 0.38 ± 0.02; Fig. 1*C*). The mGFP-FGFR3 contrast stayed within the same range regardless of the ligand addition (mean C_mGFP_ = 0.88 ± 0.01 with fgf1, Table S2), ruling out that the increase in C_mScarlet_ was a consequence of an increase of C_mGFP_. Note that ligand-induced receptor internalization is prevented in our experimental setup as mGFP-FGFR3 is bound to the surface-immobilized antibody in patterned areas (41). As a negative control, we introduced a mutation (K508M) that inhibits the transautophosphorylation of the FGFR3 kinase domain and thus recruitment of GRB2, which almost completely abolished GRB2-mScarlet colocalization (mean C_mScarlet_ = 0.05 ± 0.01) (Fig. 1*C*). Ligand addition did not induce GRB2 recruitment in cells expressing the kinase-dead mutant K508M (mean C_mScarlet_ = 0.05 ± 0.01). We also designed an mGFP-FGFR3 construct featuring a C-terminal mScarlet domain (mGFP-FGFR3wt-mScarlet) as a positive control for maximum colocalization, which yielded a mean C_mScarlet_ = 0.71 ± 0.02.

**Table 1:** Statistical parameters for all FGFR3 variants tested without ligand

Variant	Variant (CDS)	Cells	C_mScarlet_ mean ± SE	*p*	C_mGFP_ mean ± SE
K508M	c.1523A>T	56	0.05 ± 0.01	1.27E-13	0.82 ± 0.01
Positive CTR	None	36	0.71 ± 0.01	6.61E-33	0.93 ± 0
WT	None	115	0.24 ± 0.02	Not applicable	0.85 ± 0.01
G380R	c.1138G>A	51	0.42 ± 0.02	5.86E-09	0.90 ± 0.01
Y373C	c.1118A>G	49	0.43 ± 0.03	3.92E-09	0.89 ± 0.01
K650Q	c.1948A>C	56	0.50 ± 0.02	3.20E-16	0.89 ± 0.01
K650E	c.1948A>G	54	0.35 ± 0.02	2.62E-04	0.87 ± 0.01

The second column indicates the position of missense mutation in the coding sequence of FGFR3 (CDS) introduced in the expression plasmid. The *p* value represents the pairwise comparison of the respective C_mScarlet_ to the nonactivated WT. For the complete dataset also with the addition of the ligands fgf1 and fgf2, see Table S2.

Note that C_mScarlet_ values in the absence, as well as in the presence of fgf1, showed a large variability between individual cells. To ascertain that this variation in C_mScarlet_ was independent of cellular expression levels of the adaptor protein GRB2-mScarlet, we compared it with the adaptor fluorescence intensity ON (I_ON,mScarlet_) or OFF the patterned areas (I_OFF,mScarlet_). We did not find a correlation between C_mScarlet_ and I_ON,mScarlet,_ suggesting that the extent of recruitment of GRB2 to FGFR3 (expressed as C_mScarlet_) is not dependent on differences in expression levels of the transfected cells but indeed reports on the extent of receptor activation (Fig. S1). I_OFF,mScarlet_ decreases with C_mScarlet_ as expected, since OFF areas are depleted of GRB2 as it is recruited to the receptor.

### GRB2 colocalization correlates with FGFR3 activation

We next tested whether the contrast of the adaptor protein GRB2 was affected by the expression levels of the receptor. For this purpose, we examined a series of correlations of C_mScarlet_ with the density of FGFR3 in ON (I_ON,mGFP_) and OFF (I_OFF,mGFP_) regions without fgf1 addition (Fig. 1*D*). Here, we observed the following: (i) a cell population (approximately 50%) with C_mScarlet_ >0.2 showing a weak positive correlation between the GRB2 contrast and ON regions (Spearman's correlation coefficient between C_mScarlet_ and I_ON,mGFP_ of *r* = 0.37, significance level 0.004; Fig. 1, *D* and *E* and Table S1); (ii) a second population of cells of approximately 50% with C_mScarlet_ ≤0.2 (*gray area* in Fig. 1, *D* and *E*). This population was reduced when adding the ligand fgf1 (Fig. 1*D*).

We suspected the cell population with contrast levels above 0.2 to represent activated cells. Indeed, for the kinase-dead mutant (K508M), we only observed the population with C_mScarlet_ ≤0.2, likely representing the nonactivated cell population (Fig. 1, *F* and *G*). For this reason, all further correlation analyses were based on the cell population with high contrast values of C_mScarlet_ >0.2. Interestingly, even in the absence of ligand, a substantial number of cells (∼50%) showed a basal recruitment of GRB2 to the nonliganded WT receptor, possibly because of the transautophosphorylation between FGFR3s in the crowded environment inside the micropatterns. As expected though, GRB2 recruitment further increased with the addition of ligand.

Next, we compared C_mScarlet_ with I_OFF,mGFP_ as an indicator for FGFR3 expression levels (Fig. 1*E*). We did not observe a correlation neither in the absence nor in the presence of fgf1 (Table S1). This suggests that GRB2 recruitment is rather a consequence of FGFR3 activation within patterns and not just of different FGFR3 expression levels *per se.*

#### Effect of disease-relevant mutations on *FGFR3* activation

**Figure 2:**
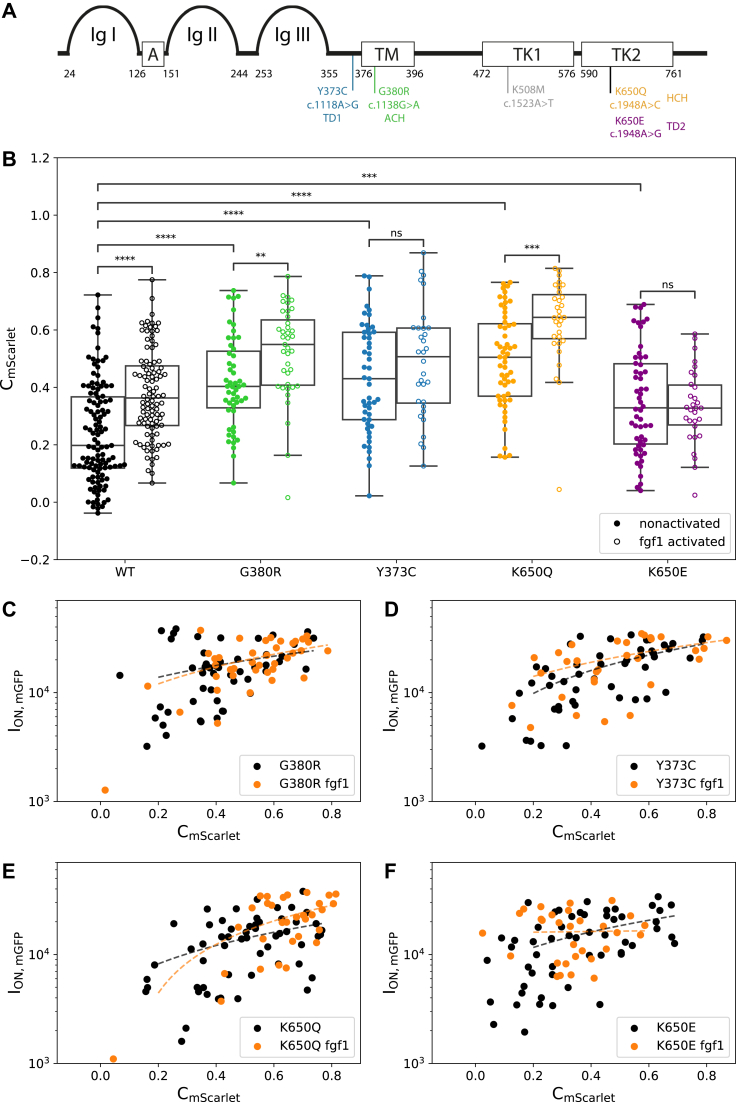
**Effect of FGFR3 mutations on receptor activation levels.***A,* schematic representation of the FGFR3 and the different tested mutations: Ig-like domains (Ig I–Ig III); acidic box (*A*), transmembrane (TM) domain, and an intracellular split tyrosine kinase domain (TK1 and TK2). Numbers indicate the amino acid position of the respective domains. Mutations are indicated at their approximate location in the protein domains with their respective amino acid and nucleotide substitutions and associated congenital disorder. *B,* comparison of the adaptor contrast (C_mScarlet_) determined for the WT and mutant forms of FGFR3 in the absence and presence of fgf1. *C*–*F,* correlation between the receptor intensity in ON areas (I_ON,mGFP_) and the adaptor contrast (C_mScarlet_) for the mutant receptors (Spearman correlation coefficients [*r*] for each mutant are listed in Table S1). The *p* value annotation legend is ∗0.01 ≤ *p* ≤ 0.05; ∗∗0.001 ≤ *p* ≤ 0.01; ∗∗∗0.0001 ≤ *p* ≤ 0.001; and ∗∗∗∗*p* ≤ 0.0001. A full list of *p* values can be found in Table S3. FGFR3, fibroblast growth factor receptor 3; Ig, immunoglobulin.

After having established that our experimental setup is sensitive enough to detect differences in GRB2 recruitment for the nonliganded and fgf1-stimulated WT receptor, we next quantified the FGFR3 activation state for four selected FGFR3 mutations (K650Q, G380R, Y373C, and K650E) described to lead to increased FGFR3 signaling (Fig. 2*A*). Each of these variants causes a different disorder with the latter being very severe and embryonic lethal: HCH, ACH, TDI, and TDII. In our experiments, all four variants exhibited a significantly higher mean C_mScarlet_ than the WT receptor without the addition of ligand, indicating the promiscuous recruitment of the adaptor protein GRB2 to the receptor without activation by the ligand (Fig. 2*B* and Table 1). Yet, all variants exhibited mGFP-FGFR3 patterns of similar contrast values (mean C_mGFP_ = ∼0.9), which did not change after fgf1 addition (Table 1 and Table S2).

The TM domain mutation G380R, which has been suggested to increase the propensity of the receptor to dimerize (45, 48, 49), showed an elevated activation level with more GRB2 colocalizing to the receptor (mean C_mScarlet_ = 0.42 ± 0.02) than the WT (Table 1 and Fig. 2*B*). Upon addition of fgf1, we observed an increase of this colocalization with a mean C_mScarlet_ = 0.52 ± 0.03 (Fig. 2*B* and Table S2) and a shift of the cell population from C_mScarlet_ >0.2 to C_mScarlet_ >0.4 (Fig. 2*C*).

Similarly, located in the extracellular (EC) juxtamembrane region, the Y373C mutation is hypothesized to induce receptor dimerization *via* disulfide bond formation causing the constitutive activation of the receptor (38, 50, 51). Cells expressing the Y373C receptor showed an intrinsic receptor activation with a high GRB2 colocalization without the addition of the ligand (mean C_mScarlet_ = 0.43 ± 0.03) as seen in Table 1 and Figure 2*B*. Compared with the G380R mutant, this mutant did not have a population of nonactivated cells with C_mScarlet_ <0.2 (Fig. 2*D*). Consistently, addition of fgf1 did not increase the activation of the receptor. Similar to the WT, for both mutants, we also observed a positive correlation between the density of FGFR3 on the pattern (I_ON,mGFP_) and C_mScarlet_ without and with the addition of the fgf1 ligand (Fig. 2, *C* and *D*). The Spearman correlation coefficient *r* was 0.28 for G380R *versus* 0.57 for Y373C (Table S1).

The mutations K650Q and K650E affect the same lysine in the kinase domain leading to strong (K650E; (52, 53)) or moderate (K650Q; (25)) constitutive receptor activation. Similar to our observations for the G380R variant, K650Q showed elevated FGFR3 activity already in the absence of ligand, which was further increased by fgf1 yielding mean contrast values of 0.50 ± 0.02 and 0.63 ± 0.03, respectively (Table 1). For this variant, we did not observe a population of nonactivated cells, neither in the absence nor in the presence of ligand (Fig. 2*E*). A weak positive correlation between C_mScarlet_ and I_ON,mGFP_ (Spearman's correlation coefficient, *r* = 0.42) was only found in the presence of ligand (Table S1).

Finally, the K650E mutation showed the lowest recruitment of GRB2 to FGFR3 (C_mScarlet_ = 0.35 ± 0.02), in spite of previous studies having reported the K650E mutation as one of the highest activating versions of FGFR3 (25, 33). While fgf1 addition did decrease the fraction of nonactivated cells (Fig. 2*F*), GRB2 recruitment was still low compared with the WT and other variants (Fig. 2*B* and Table 1). Furthermore, for K650E, we only observed a correlation between receptor density ON patterns (I_ON,mGFP_) and contrast C_mScarlet_, for the nonactivated state (Table S1).

In order to rule out any effect from differences in FGFR3 surface expression, we also tested for correlations between C_mScarlet_ and the receptor density OFF patterns (I_OFF,mGFP_) for all the analyzed mutants (Table S1 and Fig. S2) but did not find any significant correlations neither in the presence nor in the absence of fgf1.

### Effect of fgf2 on FGFR3 activation

**Figure 3:**
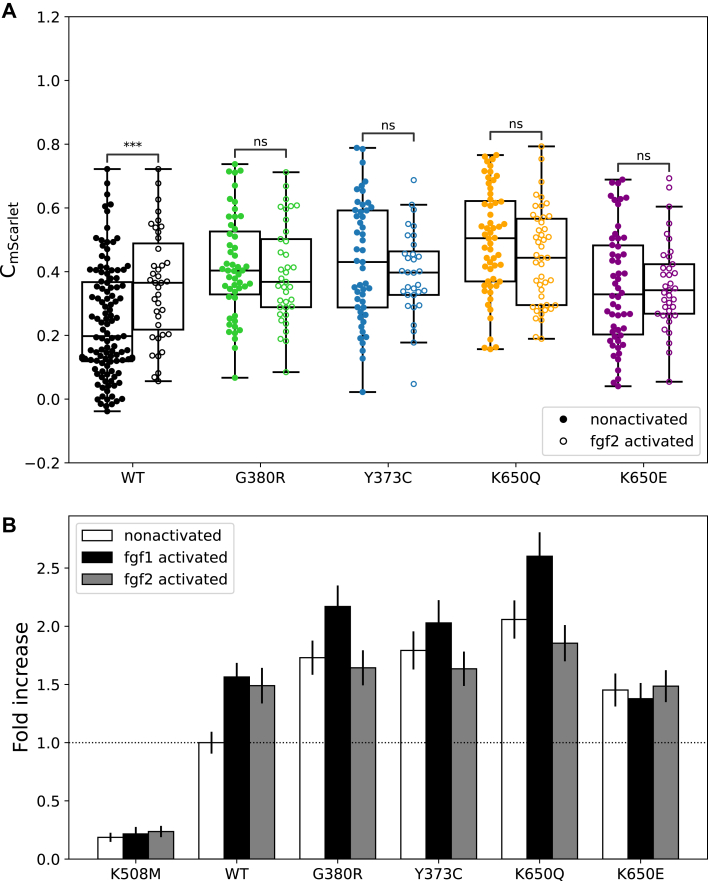
**Effect of the ligands fgf1 and fgf2 on receptor activation.***A,* comparison of GRB2 contrast (C_mScarlet_) determined for the WT and mutant forms of FGFR3 in the absence and presence of fgf2. The *p* value annotation legend is ∗0.01 ≤ *p* ≤ 0.05; a full list can be found in Table S3. *B,* normalization of mean C_mScarlet_ values to the nonactivated WT FGFR3. Data are shown as mean ± standard error. FGFR3, fibroblast growth factor receptor 3; GRB2, growth factor receptor–bound 2.

We further analyzed the different mutations using fgf2 (50 ng/ml with 1 μg/ml heparin), an alternative ligand also reported to activate the isoform IIIc of FGFR3 (8). fgf2 increased the activation of the WT receptor, albeit with a less pronounced effect than fgf1 (Fig. 3*A*, mean C_mScarlet_: nonactivated 0.24 ± 0.02 *versus* fgf2 0.36 ± 0.03). We did not detect a significant increase of mean C_mScarlet_ upon addition of fgf2 for all the mutants G380R, Y373C, K650Q, and K650E (Fig. 3 and Table S2). In short, fgf2 has a similar effect compared with fgf1 in the WT and no effect in the mutants.

In accordance with our fgf1 data, we also tested the fgf2-activated cells for correlations of C_mScarlet_ to either I_ON,mGFP_ or I_OFF,mGFP_. Similar to the fgf1 data, nonactivated cells (C_mScarlet_ <0.2) almost completely disappeared after addition of fgf2 for the WT and the mutants G380R and K650Q (Table S1 and Fig. S3) even though the mean C_mScarlet_ with fgf2 was slightly lower compared with fgf1 (Table S2). For K650Q, we also found a positive correlation between C_mScarlet_ and I_ON,mGFP_ after addition of fgf2 as well as for Y373C and K650E (Table S1). Similar to the WT or the other mutants, there was no correlation, except for G380R that shows a negative correlation between C_mScarlet_ and I_OFF,mGFP_ (Table S1).

In order to better visualize the differences in receptor activation among the different mutants, as well as their respective behavior in response to ligand addition, we normalized the mean C_mScarlet_ to the nonliganded WT receptor (Fig. 3*B*). It can be observed that the GRB2 recruitment to the WT receptor in the absence of ligand is significantly increased (approximately fivefold) compared with the negative control K508M. The addition of fgf1 or fgf2 to the WT leads to a ∼1.5-fold increase in C_mScarlet_ compared with the nonstimulated WT. All the mutants exhibit at least 1.5-fold increased GRB2 recruitment in the nonactivated state, with the K650Q mutation showing the highest increase of about twofold. The overall highest signaling can be observed in the fgf-1-activated K650Q mutant, which reaches almost 2.5 times the level of the noninduced WT.

## Discussion

### Quantification of FGFR3 signaling in live cells on micropatterned surfaces

In this work, we used a protein micropatterning approach (41, 44) to quantify FGFR3 activity in living cells. By monitoring the recruitment of a downstream signaling molecule (in our case GRB2) to FGFR3 at the plasma membrane, we directly captured the activity of the mature receptor at the cell surface, in contrast to the more commonly used methods that analyze whole-cell lysates (*e.g.*, Western blotting). This aspect is important since in this case endocytic vesicles and/or partially processed receptor in the endoplasmic reticulum or Golgi apparatus contribute to the detected signal, thus distorting the results (38, 39, 54). Note that we also excluded possible influences of FGFR3 and GRB2 expression levels as well as pattern quality by analyzing correlations between C_mScarlet_ and I_ON,mScarlet_, I_OFF,mScarlet_, I_ON,mGFP_, and I_OFF,mGFP_ in the absence and presence of ligand. We found that all four FGFR mutations tested in our study yielded in a promiscuously active surface receptor with higher levels of GRB2 recruitment than the WT even in the absence of ligand. Furthermore, we quantified the extent of activation in response to two different FGFR3 ligands, fgf1 and fgf2. We observed that ligand addition resulted in an additional increase in GRB2 recruitment for G380R and K650Q but not for the variants Y373C and K650E.

In our system, the receptor is enriched and immobilized according to specific patterns in the plasma membrane. As expected, we observed an increased recruitment of GRB2 to FGFR3-enriched areas, indicated by an increase of the contrast (mean C_mScarlet_) when adding the ligand. The recruitment was also proportional to the abundance of the receptor in the patterns (I_ON,mGFP_) (Fig. 1, *D* and *E*). Interestingly, in a subpopulation of cells, we observed already some GRB2 recruitment in the WT without the addition of the ligand. This basal recruitment might be driven by the transautophosphorylation of FGFR3s forming dimers or multimers in the crowded environment of the micropatterns. It has been described that a different RTK (epidermal growth factor receptor) can form dimers in the absence of ligand leading to ligand-independent activation (55). Moreover, this RTK has an optimal activity as an oligomer compared with the dimer form (55). Of the three FGFRs, FGFR3 has the highest intrinsic propensity for dimerization even in the absence of ligand (10). Furthermore, the receptor activity can be triggered by a higher receptor density, which is a common mechanism to increase the signaling output in cancer cells associated with FGFR upregulation (24). Thus, we hypothesize that the clustering of FGFR3 in our micropatterns enhances the receptor activation in the absence of ligand.

### TM mutants show promiscuous activation

We showed that four different FGFR3 mutations associated with human congenital disorders preactivate the signaling of the receptor at the plasma membrane. Specifically, we analyzed the FGFR3 mutations K650Q, G380R, Y373C, and K650E described to lead to increased FGFR3 signaling and different disorders of increasing severity (HCH, ACH, TDI, and TDII, respectively). The mutations Y373C and G380R are located next or within the TM domain, respectively. The Y373C variant has been described to lead to the formation of covalently bound dimers, formed by a disulfide bond between the free cysteine residues introduced by the mutation (51). Similarly, the variant G380R introduces a positively charged residue into the main dimer interface created by interactions among the residues Y379, F384, and F386 (56). Thus, also this mutation stabilizes FGFR3 dimers already in the absence of ligand, but to a lesser extent than the covalent bond in case of Y373C, and leads to a weak increase in receptor activity (33). In accordance with these observations, our study showed (i) ligand-independent activation of both mutants in the basal state, with Y373C showing a higher level of activation than G380R (Figs. 2*B* and 3*B*) and (ii) a further increase of activity upon stimulation with fgf1 only for the G380R variant (Figs. 2*B* and 3*B*). Finally, the higher activation of Y373C compared with G380R provides a plausible explanation of the phenotypic differences of TDI (caused by Y373C) with ACH (caused by G380R), with the former being often more severe.

### Activation behavior of kinase domain mutations

In our study, we did not observe a direct correlation between the activation level and the phenotypic severity for all tested mutants, as was proposed in earlier studies for other mutations of FGFR3 (25, 32, 33). For the mutations in the kinase domain (K650E and K650Q), the activation behavior of the mutant receptors was more complex. The K650E variant changes the conformation of the tyrosine kinase domain similar to ligand-mediated FGFR3 dimerization and autophosphorylation (52, 53). The K650Q variant has been described to also result in constitutive kinase activation; however, to a lesser degree than the K650E mutant (25). In contrast, we found that the high basal level of activity of the K650Q variant could be enhanced even further by the addition of fgf1.

Pertaining to the phenotypic consequences of the mutation, K650Q is associated with HCH, which is considered a milder form of ACH (22), and also milder than the other skeletal dysplasias TDI and TDII. Thus, this particular example (K650Q) with the highest promiscuous activation measured in our system (approximately twofold higher than WT) does not follow the expectation that the severity of the skeletal dysplasia is correlated with the degree of the activation of the mutant receptor.

Another intriguing observation is that, contrary to previously published data, the kinase mutation K650E had the lowest GRB2 recruitment of all four analyzed mutations (Figs. 2*B* and 3*B*), although it has been described as one of the mutations leading to the highest constitutive receptor activation (33, 52, 53). A partial, and maybe still unsatisfactory, explanation for these contradictory observations might be the cellular compartment harboring the mutant protein. Previous studies have shown that mutant forms of the FGFR3 IC protein chain are not released and remain inside the cellular export machinery (38, 39, 54). While these IC fractions can still induce basal activation of the downstream extracellular signal–regulated kinase proteins (38, 39, 54), the biological importance of these immature receptors is still unclear. Studies analyzing the activation in whole-cell lysates (25, 33, 53) would capture the receptor in the IC compartments and in the cell membrane. Our system specifically reports on signaling events happening at and/or near the plasma membrane and thus responding to ligand induction.

### fgf1 *versus* fgf2 ligand activation

In this study, we determined differences between the fgf1 and fgf2 ligands for recruiting the adaptor GRB2. Using the micropatterning approach, we observed that fgf1 leads to similar GRB2 recruitment to the WT receptor. However, the addition of fgf2 did elicit a weaker activation to the different mutant forms (Figs. 2*B* and 3*B*), which could be explained with previous findings showing that fgf1 has a higher affinity for FGFR3 isoform IIIc (also used in our study) and binds more stably than fgf2. However, fgf2 leads to higher FGFR3 kinase phosphorylation at saturating concentrations (above 1 μg/ml), when tested *via* phospho-specific Western blotting (57). Similar results were obtained when using a truncated form of FGFR3 and highly saturating conditions for both fgf1 and fgf2 (5 μg/ml), showing that fgf1 induced a lower phosphorylation activity than fgf2 (10). The reported physiologic abundance of the ligands detected in plasma of healthy individuals is approximately 4 ng ml^−1^ for fgf1 (58) and at approximately 107 pg ml^−1^ for fgf2 (59). Although we used ligand concentrations far from these physiological conditions (50 ng/ml fgf1 or fgf2), we hypothesize that the binding strength of each ligand might control better the signaling output of the receptor at nonsaturating conditions than under saturating conditions. In addition, fgf1 is less stable compared with fgf2 (60), so it is difficult to estimate the effective ligand concentration in our system. Furthermore, we also used heparin that has also a strong stabilizing effect on fgf1 but a more moderate effect on fgf2 (60).

In conclusion, the effect of mutations on the activation behavior of FGFR3 is diverse; as reported here, some mutants are still responsive to ligand binding, but others already result in a very high promiscuous receptor activity and do not further increase their activity upon ligand addition (*e.g.*, Y373C compared with G380R), which is of importance for therapies using ligand inhibitors. The method we present here complements existing approaches in that it allows to test these activation differences directly at the plasma membrane in living cells and measure the response to the ligand.

## Experimental procedures

### Cloning and plasmids

The aim was to create a fusion construct containing the coding sequence of the human *FGFR3* gene (isoform 1, also known as *FGFR3-IIIc*) with an N-terminal mGFP tag. The sequence of the mGFP contains a mutation that prevents dimerization of the fluorophore (61). The final fusion protein was cloned into the pcDNA3.1/Hygro(+) vector (Thermo Fisher Scientific), suitable for expression in mammalian cells. For proper IC transport and secretion of the fusion protein, the signal peptide (SP) was cloned N terminal of the mGFP, followed by a 5xGGS-linker that serves as a spacer between the GFP and the other domains of FGFR3 (EC domain, TM domain, and IC domain); FGFR3(SP)-mGFP-5xGGS-FGFR3 (EC–TM–IC). To ensure efficient translation initiation, the strong Kozak sequence “GCCACC” (62) was cloned upstream of the “ATG”-start codon. Cloning was performed by restriction enzyme digest and ligation in two steps. In step 1, the FGFR3 domains without the SP (all primer sequences used in this study are shown in Table S4) were cloned into the empty pcDNA3.1/Hygro(+) vector, and in step 2, the generic construct FGFR3(SP)-mGFP-5xGGS, which was synthesized and cloned by BioCat (the sequence is shown in Table S5), was cloned upstream of FGFR3. A detailed description of the different steps of the cloning procedure is provided in the supporting information.

In order to introduce the specific single-point mutations studied in this article, the FGFR3 expression vector was amplified using a high-fidelity polymerase, either Q5 (NEB) or Phusion HS II (Thermo Fisher Scientific). Specifically designed (back-to-back) primers, where one primer per primer pair carries a 5′-phosphate modification, were used for the amplification, thereby introducing site-directed base changes that create nonsynonymous (and in addition several silent) mutations within the *FGFR3* gene. The silent mutations were inserted in close proximity to the actual mutation to detect potential aerosol contaminations of these plasmids in ultrasensitive sequencing technologies used in other projects in our laboratory. To avoid potential side effects because of different codon usage, the silent mutations were designed such that codons with a similar usage frequency in humans were chosen. A list of all mutated plasmids generated for this study is shown in Table S6. Description of the detailed mutagenesis procedure is given in supporting information. The expression of all FGFR3 constructs was verified in HeLa cells *via* Western blot (Fig. S4).

To obtain the GRB2-mScarlet plasmid, we carried out PCR to amplify the *mScarlet* sequence from the ITPKA-mScarlet plasmid (Addgene) as well as the *GRB2* sequence from GRB2-YFP (gift from J. Weghuber) with >15 nt overhangs complementary to adjacent regions on the target plasmid. We then used the Gibson assembly Master Mix (E2611; NEB) following the supplier’s instructions to insert both fragments into a pcDNA3.1 vector.

The positive CTR plasmid was created by PCR amplification of the complete vector containing the WT mGFP-FGFR3 expression construct using the high-fidelity polymerase Q5 (NEB). Specific primers were designed to delete the stop codon of *FGFR3* and to also add the recognition sequence for the AgeI restriction enzyme. In another PCR using Q5, *mScarlet* was amplified from our GRB2-mScarlet plasmid using primers that also contained the AgeI recognition sequence as well as additional bases that are translated into “GS” in order to generate a GGS-linker in between the FGFR3 and the mScarlet. Description of the detailed cloning procedure is given in supporting information.

We also tested the recruitment of a different adaptor protein SHC (Fig. S5). pcDNA3.1-SHC-monomeric red fluorescent protein (mRFP) plasmid was obtained as a kind gift from Peter Lanzerstorfer (FH Wels).

### Cells and reagents

HeLa cells (human cervical cancer cells, ACS7005; Sigma–Aldrich) were cultured in Dulbecco’s modified Eagle's medium supplemented with 10% fetal bovine serum, 2 mM l-glutamine, 1000 U/ml penicillin–streptomycin (all from Sigma–Aldrich) in a humidified atmosphere at 37 °C and 5% CO_2_. Cells were cotransfected with one of the pcDNA3.1-mGFP-FGFR3 construct and the pcDNA3.1-GRB2-mScarlet (or pcDNA3.1-SHC-mRFP) in Opti-MEM I reduced serum media (Thermo Fisher Scientific) using TurboFect Transfection Reagent (Thermo Fisher Scientific), according to the manufacturer’s protocol. The imaging buffer used for microscopy consisted of Hank’s balanced salt solution (Sigma–Aldrich) supplemented with 2% fetal bovine serum. FGFR3-expressing cells were activated using FGFR ligands fgf1 or fgf2 (Bio-Techne).

### Surface preparation and activation of patterned cells

Micropatterned surfaces were produced as previously published (42). In brief, polydimethylsiloxane stamps featuring circular pillars with diameters of 3 μm and a center-to-center distance of 6 μm were rinsed with absolute ethanol and dH_2_O and dried in a nitrogen flow followed by 15 min incubation with 50 μg/ml streptavidin (Sigma–Aldrich) in PBS (Sigma–Aldrich). After incubation, the polymer stamps were rinsed with dH_2_O, dried in a nitrogen flow, then printed on an epoxy-coated coverslip (Schott), pressed slightly to ensure good contact between the surfaces, and incubated for 60 min at room temperature. After stamp removal, a Secure-Seal hybridization chamber (Grace BioLabs) was placed on top of the streptavidin pattern and filled with 50 μg/ml fibronectin (Sigma–Aldrich) in PBS. After 30 min, fibronectin was removed, and 10 μg/ml biotinylated anti-GFP (Novus) in PBS with 1% bovine serum albumin (Sigma–Aldrich) was added for 30 min, followed by rinsing with PBS. For micropatterning experiments, cells were harvested approximately 24 h after the transfection using Accutase (Sigma–Aldrich), seeded onto micropatterned surfaces, and incubated for 30 min at 37 °C. Directly before the measurement, the medium was replaced with imaging buffer. After experiments with cells in the nonactivated state, the hybridization chamber was filled with imaging buffer supplemented with 1 μM heparin and incubated at 37 °C for 3 min. Then the solution in the chamber was exchanged to imaging buffer with 50 ng/ml fgf1 or 50 ng/ml fgf2, and the sample was incubated for 15 min at 37 °C. Different activation conditions (different concentrations and the presence/absence of heparin) were also tested for both ligands for the WT receptor (Fig. S6). Only for fgf1, when using half of the concentration (25 instead of 50 ng/ml), we found a significant decrease in FGFR3 activation. Although the addition of heparin had no effect on the receptor activation for neither of the ligands, possibly because of the expression of HSPG2 in HeLa cells, we included heparin in the activation to ensure equal heparin concentrations among experiments (independent of the endogenous heparin expression).

### TIRF microscopy

TIRF microscopy experiments were performed on a home-built system based on a modified inverted microscope (Zeiss Axiovert 200) equipped with 100× oil-immersion objective (Zeiss Apochromat NA1.46). mGFP was excited using a 488 nm diode laser (ibeam-smart; Toptica), and mScarlet/mRFP was excited using a 561 nm diode laser (Obis; Coherent). Laser lines were overlaid with an OBIS Galaxy beam combiner (Coherent). Emission light was filtered using appropriate filter sets (Chroma) and recorded on an iXon DU 897-DV EM-CCD camera (Andor). TIRF illumination was achieved by shifting the excitation beam in parallel to the optical axis with a mirror mounted on a motorized movable table.

### Data evaluation of contrast analysis

Images of patterned cells were analyzed using Fiji (63) as described in Ref. (42). Briefly, selection masks defining “ON” and “OFF” were determined based on the FGFR3-mGFP pattern and applied on the images recorded in the GRB2-mScarlet channel. All “ON” and all “OFF” areas of one cell (usually 4–9) were pooled, and the background-corrected mean pixel intensity values of “ON” and “OFF” regions, I_ON_ and I_OFF_, were used for further analysis. An image recorded without any illumination was used to determine the background intensity (I_bg_) of our system. The contrast value was then determined separately for each cell and color channel *via*

$$C=\frac{I_{ON}-I_{OFF}}{I_{ON}-I_{bg}}$$

Note that the background of cytosolic but membrane-proximal GRB2-mScarlet is subtracted as I_OFF_ in the calculation of the contrast. For each experiment, we collected at least 30 individual data points (cells) from at least three independent measurements (transfections) merged for the contrast plots, and the corresponding mean values were compared using a one-way-ANOVA. Exact cell numbers are presented in the respective tables. Correlation between GRB2 contrast and GFP ON pattern intensity was determined by Pearson’s correlation coefficient. Statistical analysis and plotting were implemented in Python 3, using the Numpy, SciPy, and Pandas packages for general numerical computations, Matplotlib and Seaborn for plotting, and Statannot for statistical annotation in plots.

The authors declare that they have no conflicts of interest with the contents of this article.

## Data availability

All data produced are available in this article as supporting information.

## Supporting information

This article contains supporting information.

## Conflict of interest

The authors declare that they have no conflicts of interest with the contents of this article.
