# Supplementary figures and images for: Measurement of FGFR3 signaling at the cell membrane via total internal reflection fluorescence microscopy to compare the activation of FGFR3 mutants
Source: J Biol Chem. 2022 Dec 27;299(2):102832. doi: 10.1016/j.jbc.2022.102832 (PMC9900515; doi:10.1016/j.jbc.2022.102832)

**a**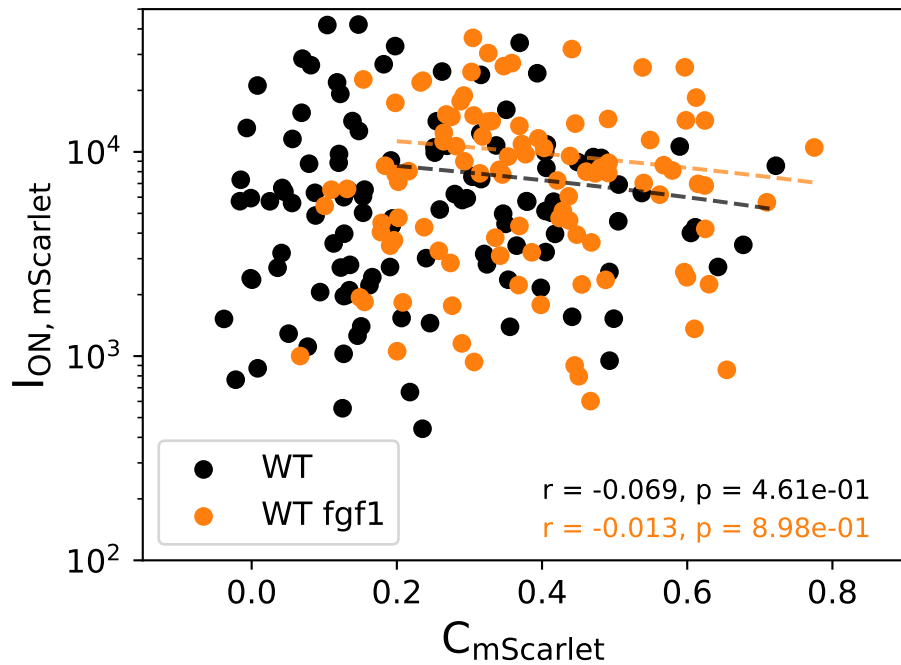**b**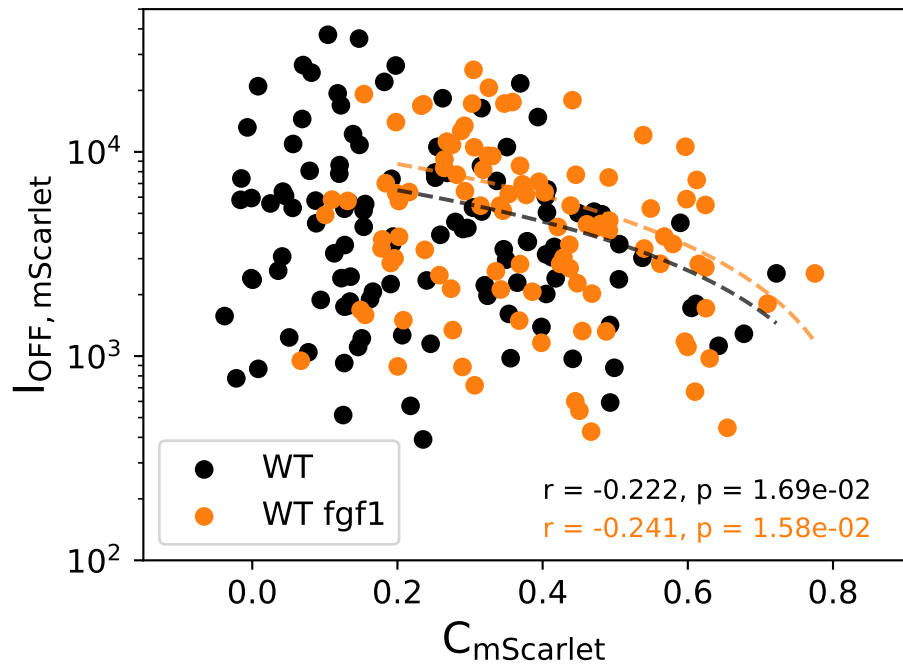

Supplement: Supplemental Figure S1 [file mmc7.pdf]

**a**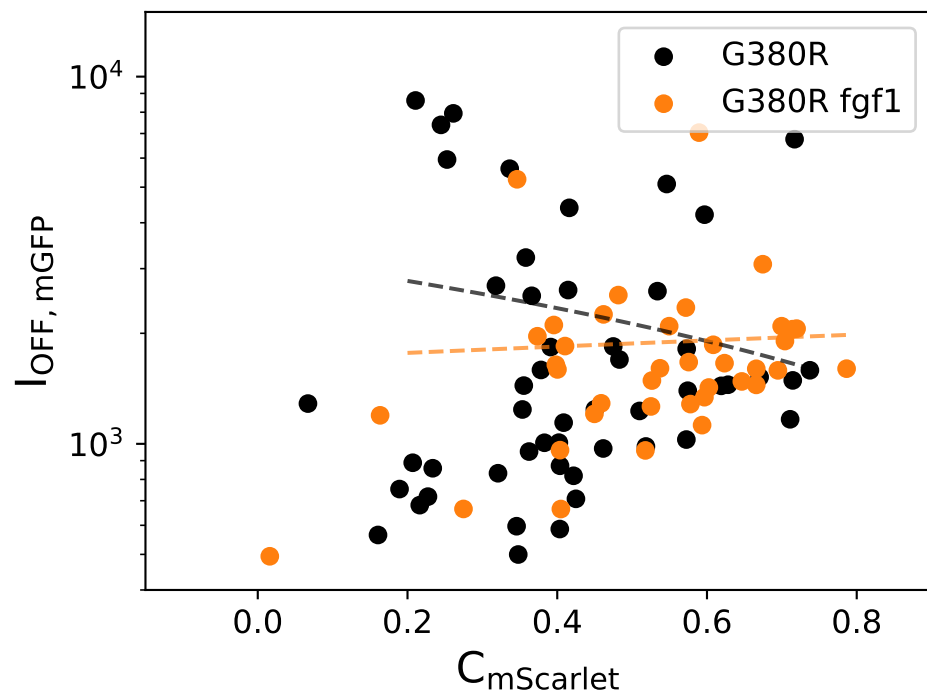**b**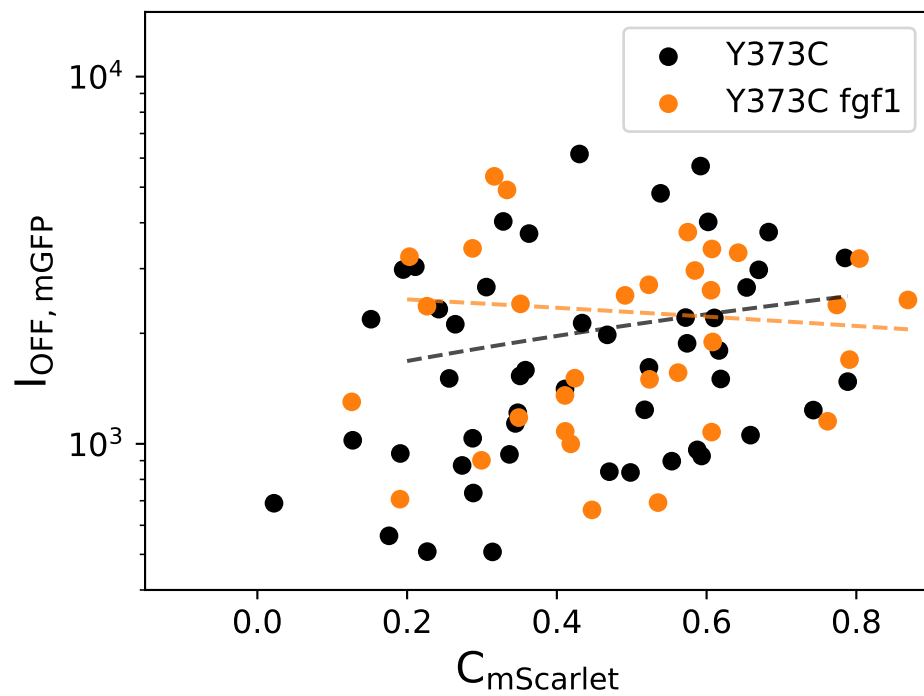**c**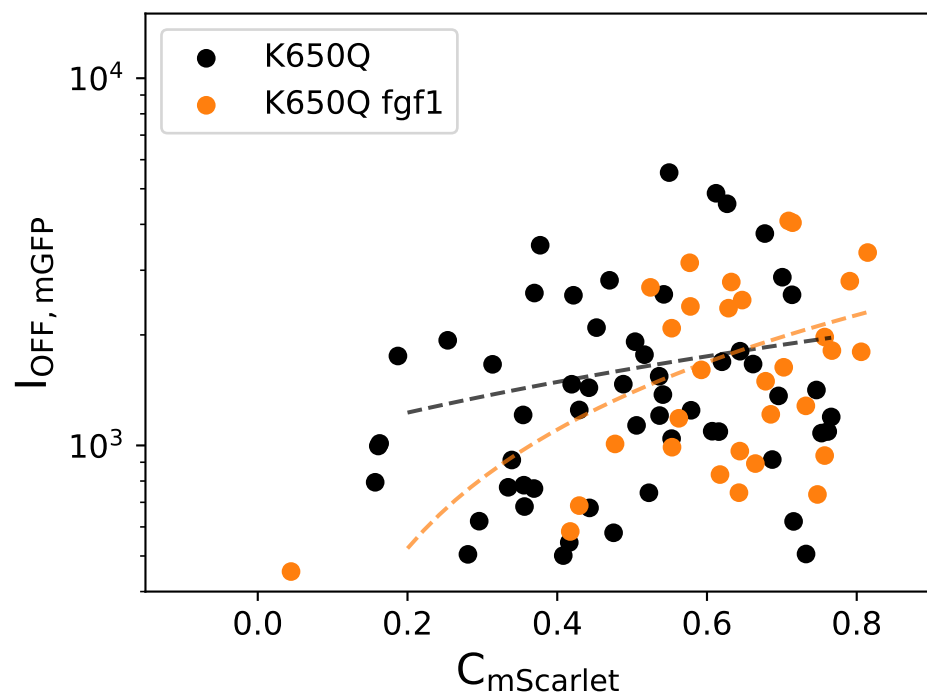**d**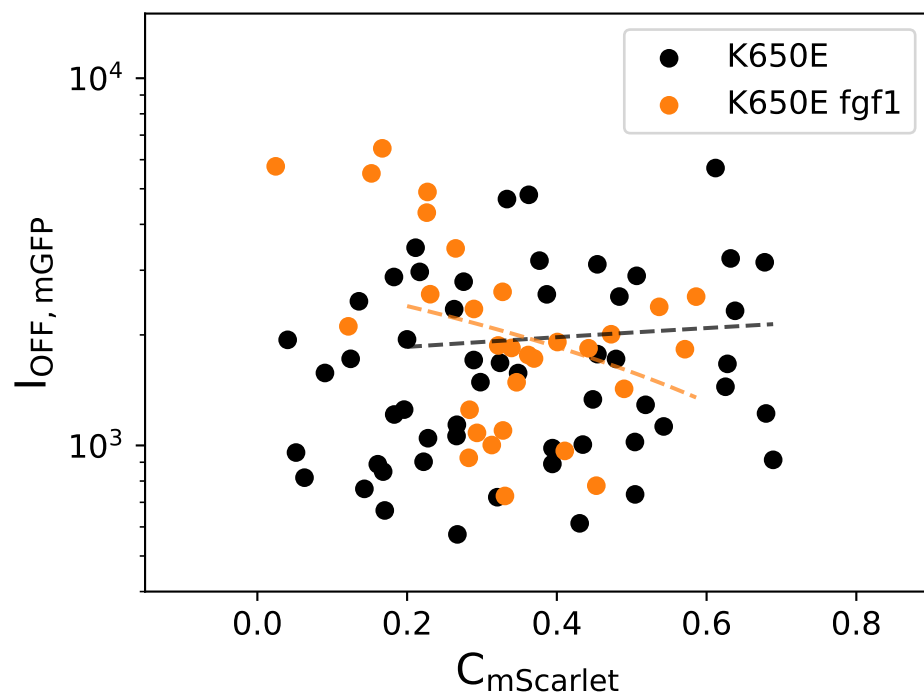

Supplement: Supplemental Figure S2 [file mmc8.pdf]

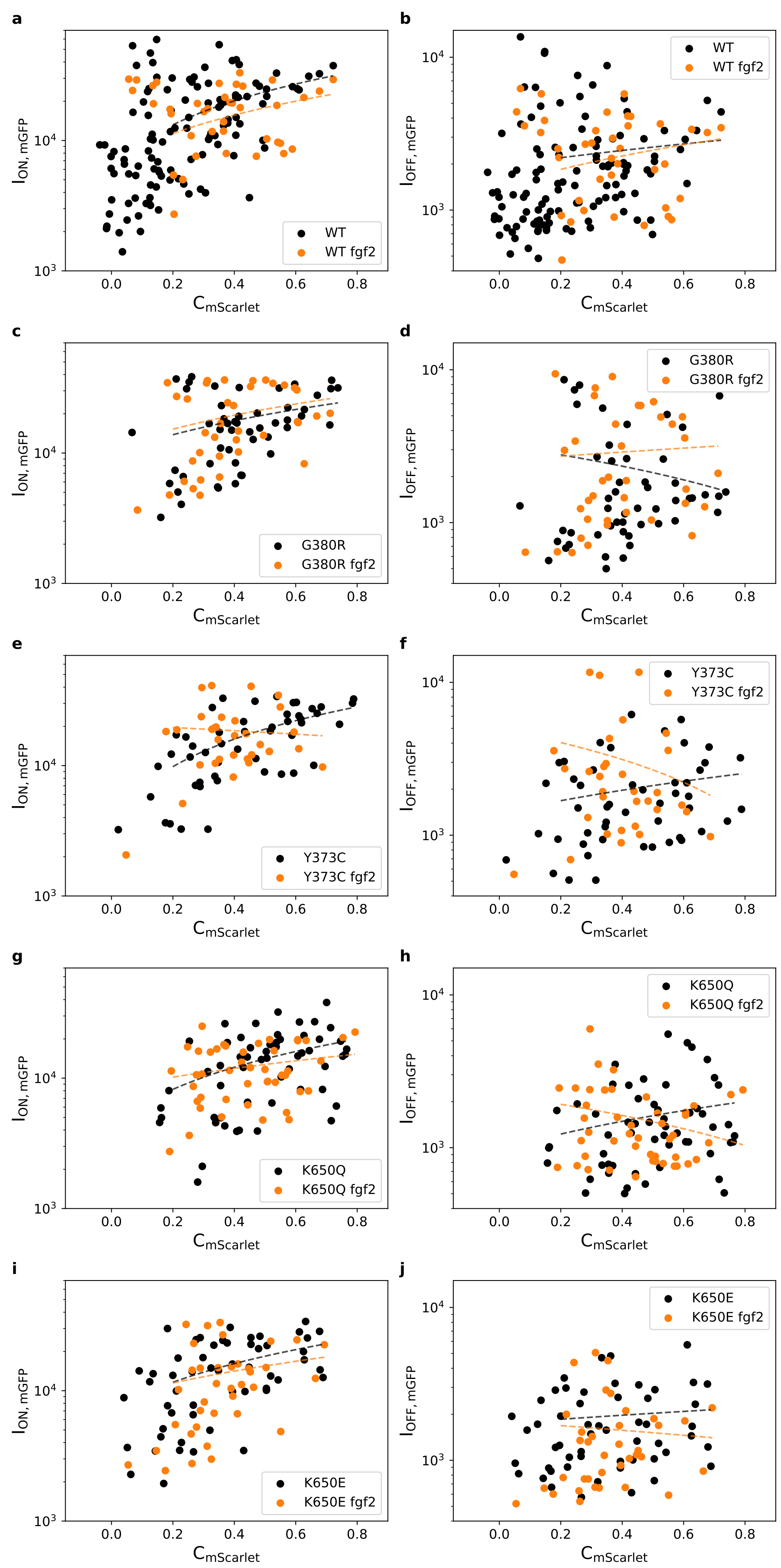

Supplement: Supplemental Figure S3 [file mmc9.pdf]

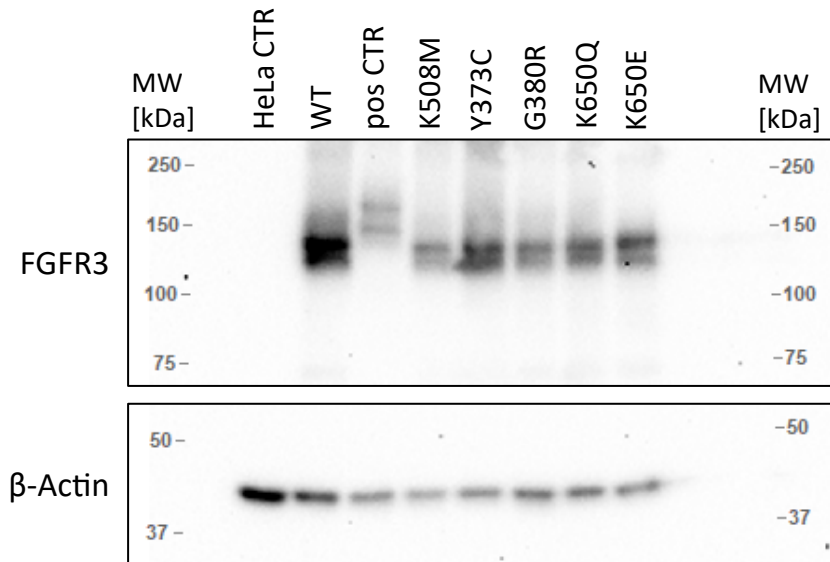

Supplement: Supplemental Figure S4 [file mmc10.pdf]

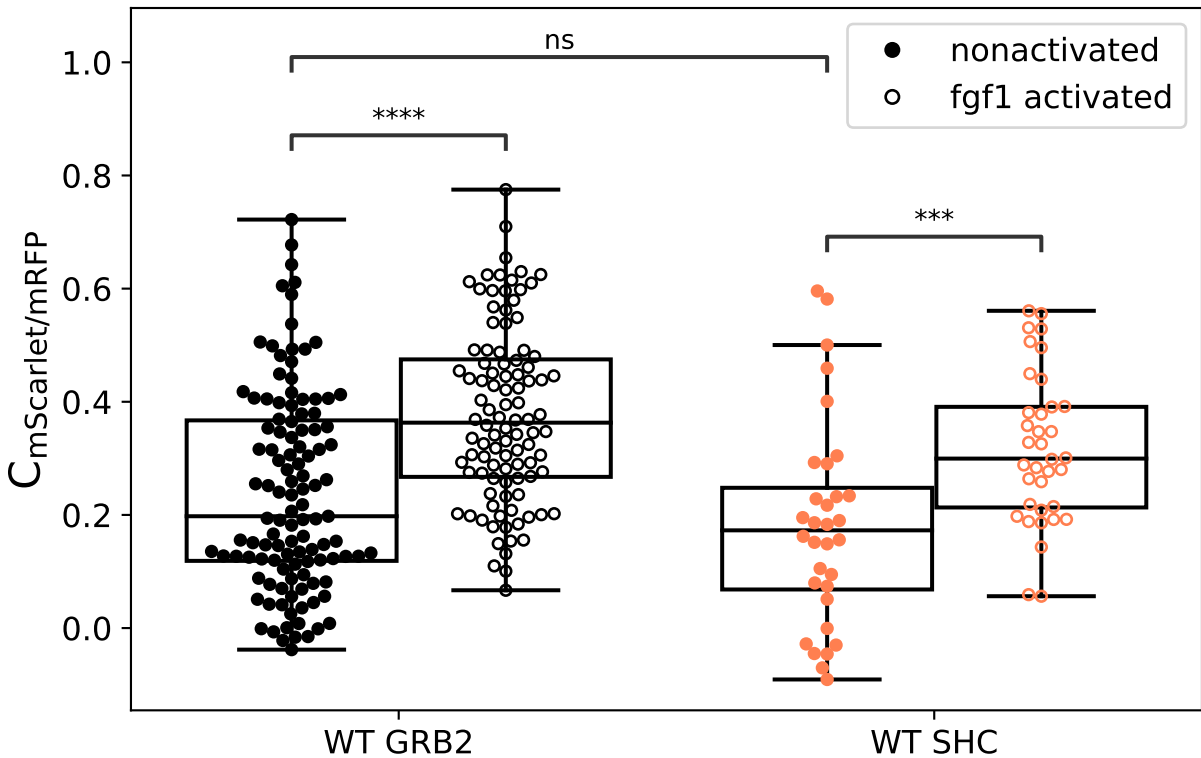

Supplement: Supplemental Figure S5 [file mmc11.pdf]

**a**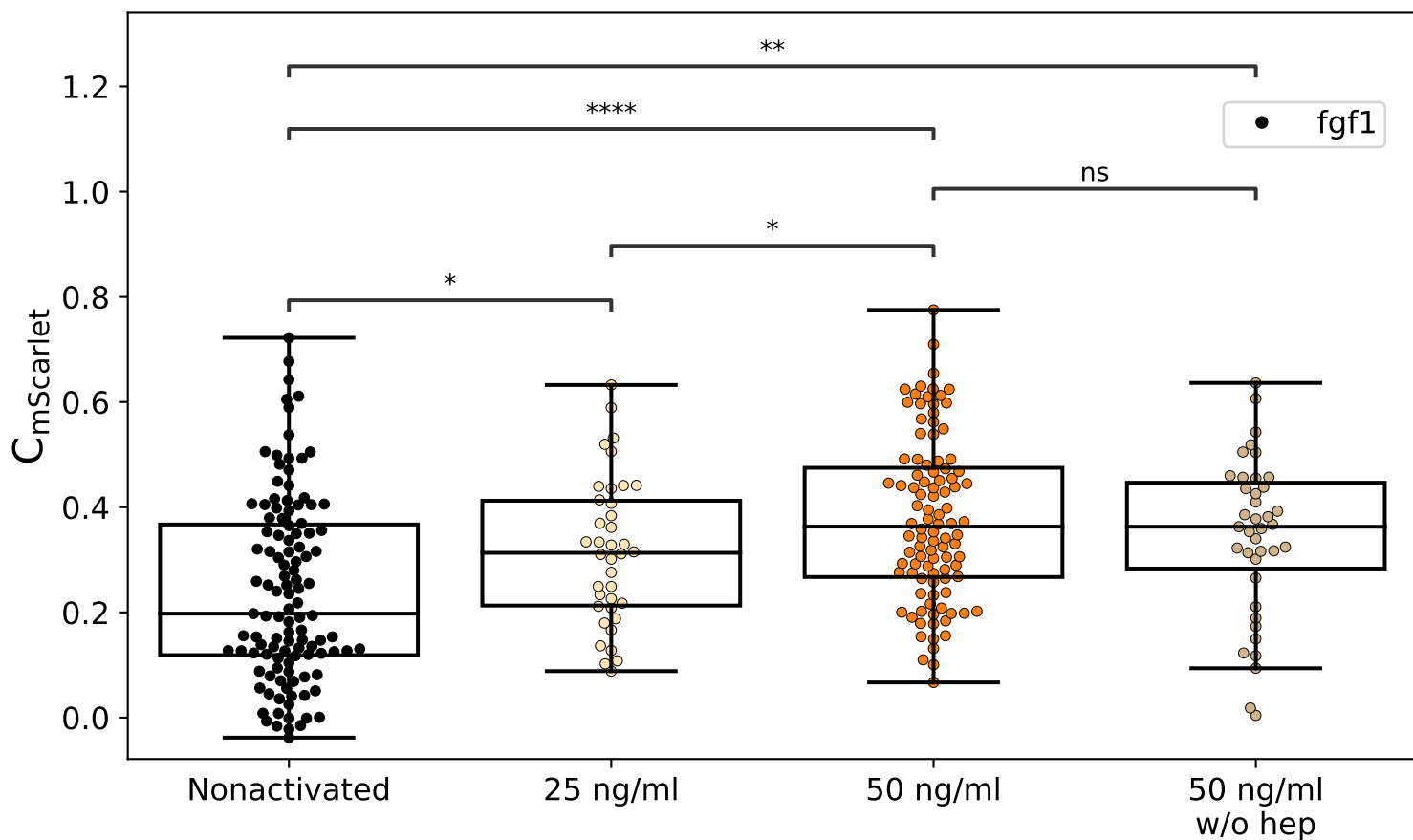**b**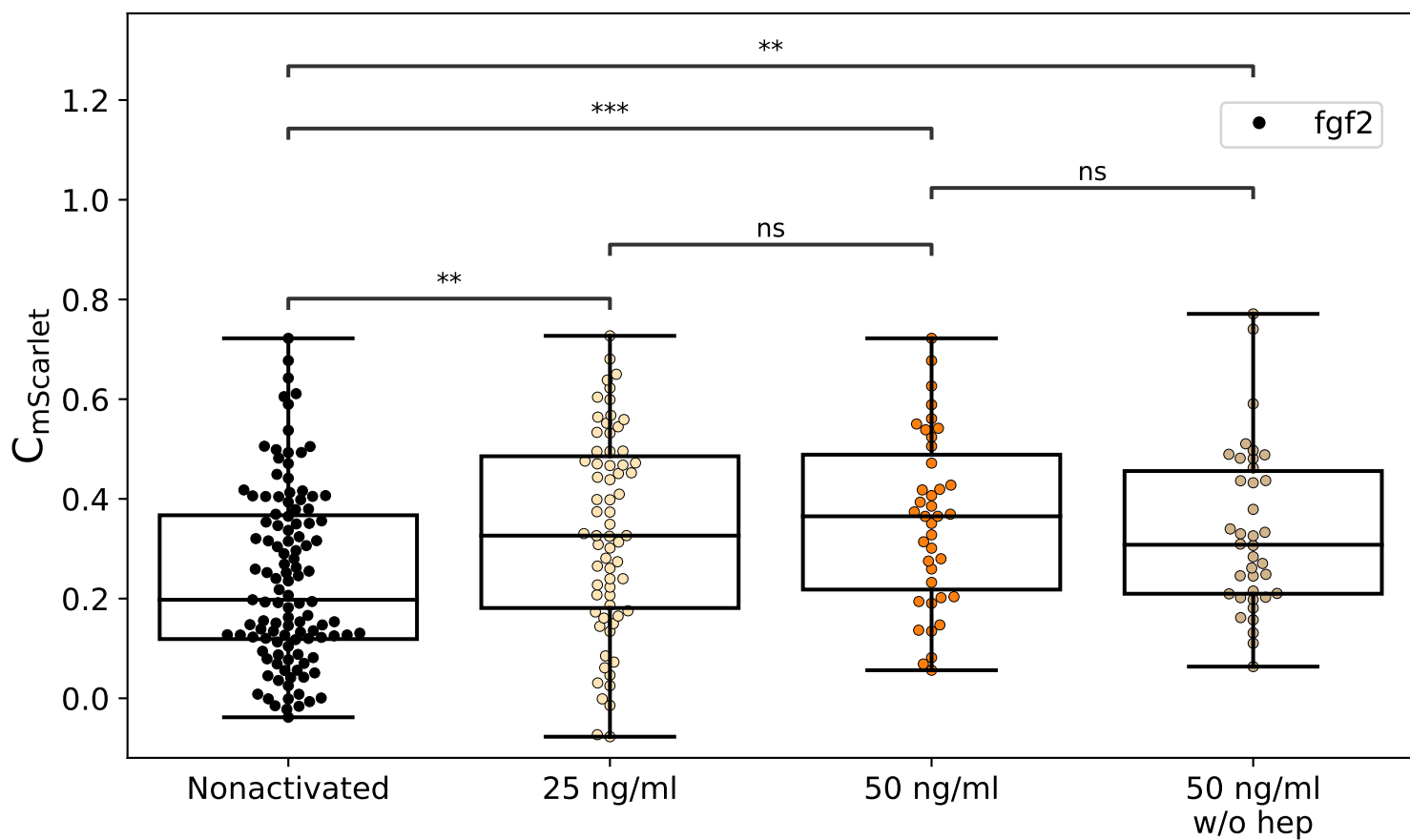

Supplement: Supplemental Figure S6 [file mmc12.pdf]
