# Supplementary material for: Measurement of FGFR3 signaling at the cell membrane via total internal reflection fluorescence microscopy to compare the activation of FGFR3 mutants
Source: J Biol Chem. 2022 Dec 27;299(2):102832. doi: 10.1016/j.jbc.2022.102832 (PMC9900515; doi:10.1016/j.jbc.2022.102832)
Supplement: Supplemental Figure Legends [file mmc13.docx]

# *Measurement of FGFR3 signaling at the cell membrane via total internal reflection fluorescence microscopy to compare the activation of FGFR3 mutants*

Ingrid Hartl^1*^, Veronika Brumovska^2*^, Yasmin Striedner^1^, Atena Yasari^1^, Gerhard J. Schütz^2^, Eva Sevcsik^2^, Irene Tiemann-Boege^1^

^1^Institute of Biophysics, Johannes Kepler University, Linz, Austria

^2^Insitute of Applied Physics, TU Wien, Vienna, Austria

^*^equal contribution

# Supplementary Figure Legends

Figure S1: Correlation analysis of GRB2-mScarlet contrast (C_mScarlet_) and intensity for WT FGFR3 before (black) and after (orange) the addition of fgf1 in (a) ON areas (I_ON,mScarlet_) and (b) OFF areas (I_OFF,mScarlet_). Pearson's correlation coefficient (r) is shown as the dotted line in the respective color.

Figure S2: Correlation between the mGFP-FGFR3 intensity in OFF regions (I_OFF,mGFP_) and GRB2-mScarlet contrast (C_mScarlet_) for G380R (a), Y373C (b), K650Q (c) and K650E (d). Data in the absence (black) and presence (orange) of fgf1 is shown. Pearson's correlation coefficient (r) is shown as the dotted line in the respective color. The values of (r) are reported in Supplemental Table 1.

Figure S3: Correlation between mGFP intensity in ON (I_ON,mGFP_) (left panel; a, c, e, g and i) and OFF regions (I_OFF,mGFP_) (right panel; b, d, f, h and j) and GRB2-mScarlet contrast (C_mScarlet_) for the WT and all the analyzed mutants. Data in the absence (black) and presence (orange) of fgf2 is shown. Pearson's correlation coefficient (r) is shown as the dotted line in the respective color. The values of (r) are reported in Supplemental Table 1.

Figure S4: Western Blot analysis of expression of FGFR3 constructs in HeLa cells. Labels above each lane indicate the plasmid used for transfection of cells, whereas HeLa CTR corresponds to non-transfected cells. Numbers on the left and right indicate the molecular weight marker bands. Note the upward shift of the positive control (pos CTR) due to the additional C-terminal mScarlet-tag in this construct. Approximately 24h after transfection, HeLa cells were directly lysed in 2x Laemmli buffer (4% sodium dodecyl sulfate (SDS), 20% glycerol, 0.0004% bromophenol blue and 2 mM dithiothreitol in 125 mM Tris-HCl, pH 6.8) and denaturated for 5 min at 95°C. Proteins were separated using an in-house prepared 8% SDS gel and afterwards transferred onto a polyvinylidene fluoride (PVDF) membrane under wet conditions. After transfer, unspecific binding sites were blocked by incubating the membrane for 60min at room temperature (RT) in 5% skim milk in TBS-T (1x Tris-buffered saline containing 0.05% Tween-20). The membrane was then cut above the height of the 50 kDa marker band and the higher molecular weight part was incubated for 1h at RT with anti-FGFR3 (used at 1:2000 in blocking solution, clone C51F2, rabbit, Cell Signaling Technologies); whereas, the lower molecular weight part was incubated with anti-β-Actin (used at 1:500 in blocking solution, clone C4, mouse, Santa Cruz). Following the incubation with the primary antibodies, the membrane parts were washed in TBS-T (3x buffer change for at least 5min incubation each) and probed with their corresponding HRP-conjugated secondary antibodies (anti-rabbit-HRP and anti-mouse-HRP, Cell Signaling Technologies). After another 3x washing in TBS-T, Pierce^TM^ ECL Western Blotting Substrate (ThermoScientific) was applied to the membrane parts and the chemiluminescence signal was detected on a ChemiDoc MP (Bio-Rad).

Figure S5: Comparison of different FGFR3-docking proteins as activation reporters. SHC-mRFP shows similar increase in mRFP contrast (C_mRFP_) upon activation with fgf1 ligand as GRB2-mScarlet.

Figure S6: The effect of different activation conditions for fgf1 (a) and fgf2 (b). WT receptor was activated using either 25 ng/ml or 50 ng/ml of ligand together with pre-incubation with heparin or using 50 ng/ml of ligand without heparin.
